# Knowledge-driven or motivation-driven? Validation and comparison of health participation pathways across different elementary grade levels

**DOI:** 10.3389/fpubh.2026.1772064

**Published:** 2026-02-18

**Authors:** Yuxing Wang, Jiayue Guo, Jianing Dai, Lizhu Liu, Mengyu Li, Ziqing Zan, Lili You

**Affiliations:** School of Health Policy and Management, Chinese Academy of Medical Sciences and Peking Union Medical College, Beijing, China

**Keywords:** chain mediation model, elementary education, knowledge, health literacy, motivation, health literacy framework, health participation

## Abstract

**Background:**

Childhood and early adolescence are critical periods for promoting health literacy (HL), a foundational factor that influences health-promoting participation throughout life. Evidence-based, theory-informed interventions to improve participation in school-aged children are scarce. This study aims to clarify the HL framework and to understand its role in guiding tailored interventions.

**Methods:**

Within the HL framework, this study conceptualized health participation improvement through knowledge, skills, and motivation. Two pathways, knowledge-driven and motivation-driven, were proposed and validated for their relative contributions. From January to July 2023, a multistage cluster sampling method was used to recruit 3,325 elementary school students in China, including 1,070 students in Grades 1–2, 1,190 in Grades 3–4, and 1,065 in Grades 5–6. HL was assessed using the Chinese Rapid Health Literacy Questionnaire (CRHLQ) for Elementary School Students, a validated assessment tool comprising three sub-questionnaires. Chain mediation models were constructed to examine the two pathways, with gender, academic performance, and personality included as control variables. Indirect effects were estimated using a bootstrap method with 5,000 replications.

**Results:**

In Grades 1–2, health participation is primarily mediated through motivation and skills for the knowledge-driven pathway, while motivation exerts a strong direct effect in the motivation-driven pathway. In Grades 3–4, knowledge has a total direct effect on health participation, whereas motivation influences participation indirectly via knowledge. In Grades 5–6, the knowledge-driven pathway operates mainly through motivation, with no direct effect, while motivation again shows a strong direct effect on participation. To sum up, pathways for improving health participation differ across elementary grade levels: motivation-driven in lower grades, knowledge-driven in middle grades, and combined knowledge- and motivation-driven in upper grades.

**Conclusion:**

Guided by the HL framework, patterns of health participation improvement differ across grade levels. HL interventions should be tailored using developmentally appropriate strategies and tools. Adapting health education content to grade level, balancing motivation-driven and knowledge-driven strategies, can maximize its educational effectiveness.

## 1 Introduction

Children worldwide are increasingly exposed to complex and rapidly evolving health risks, including overweight and obesity, declining vision, insufficient physical activity, unhealthy dietary patterns, mental health problems, excessive digital media use, and pervasive sleep difficulties (1, 2). These behavioral risk factors not only impede academic achievement and social development but also substantially increase the long-term risk of non-communicable diseases (3). The COVID-19 pandemic has worsened these vulnerabilities and increased disparities.

Improving health literacy (HL) among this population has become a global priority. HL is commonly defined as the ability to access, understand, appraise, and apply health information to inform decision-making in healthcare, disease prevention, and health promotion contexts (4, 5). Contemporary frameworks conceptualize HL as a multidimensional and developmental construct that integrates knowledge, practical skills, critical appraisal, and motivation (6). In children, it manifests through age-related changes in cognitive abilities, literacy skills, and health-related behaviors (7). The elementary school years represent a critical period for HL development, as children begin to transition toward greater autonomy and responsibility for their own health (8). Younger children may rely more on interest, whereas older children may depend more on knowledge and integrated skills (9–11). HL is associated with health issues such as obesity, psychological distress, and harmful digital behaviors (4). The WHO Health-Promoting Schools (HPS) approach, and HL initiatives led by the EU and OECD, recognize the development of HL competencies as fundamental to children’s health and well-being (12). In China, HL was embedded within Healthy China 2030 (13, 14), the Compulsory Education Curriculum Standards (15), and the Core Competencies for Chinese Students’ Development (16).

HL is a core outcome of school health education, and HL frameworks and assessments guide the health promotion interventions. In the field of health promotion, two major pathways have long coexisted: knowledge-driven and motivation-driven pathways. The knowledge-driven pathway, grounded in theories such as the Health Belief Model, posits that behavior change is primarily initiated through cognitive understanding of health risks and benefits. Social Cognitive Theory highlights the importance of self-efficacy and positions skills as essential mediators between knowledge and action (17). The motivation-driven pathway, rooted in frameworks such as Self-Determination Theory, prioritizes fostering intrinsic motivation and fulfilling basic psychological needs (e.g., autonomy, competence) as essential for sustained behavior change (18).

Accordingly, in health promotion practice, the emphasis placed on knowledge and motivation often differs (19, 20). Systematic reviews across nutrition, physical activity, and mental health education show that programs integrating skills training with motivational components yield stronger behavioral outcomes than purely informational approaches (21, 22). A recent quasi-experimental study among adolescents emphasized that motivational factors mediate the translation of health learning into actual behavior (23).

In real-world educational settings with constrained resources, educators often must make choices in allocating limited resources between knowledge instruction and fostering motivation. In this study, we defined HL as a multidimensional construct and conceptualized health participation improvement through knowledge, skills, and proposed two pathways: “knowledge-driven” and “motivation-driven”. By empirically examining the two pathways, this study aims to clarify how the HL framework guides health participation development across different elementary grade levels and generate insight for dynamic, stage-specific interventions.

## 2 Materials and methods

### 2.1 Study design and participants

To examine the two proposed pathways, a cross-sectional study was conducted from January to July 2023 using a multistage cluster sampling method. In the first stage, four provinces spanning the southern, central, and northern China were selected: Sichuan, Henan, Shanxi and Beijing. In the second stage, one city was randomly selected from each division, specifically Luzhou, Zhengzhou, Taiyuan and Beijing. In the third stage, two primary schools were selected within each sampled city. In the final stage, one to two classes were randomly sampled from each of the three grade levels (Grades 1–2, 3–4, and 5–6) at each school. Data were collected using a structured health literacy questionnaire (described below), which was administered to all students. Ethical procedures followed a strict dual-consent process: written informed consent was obtained from parents or legal guardians, and assent was obtained from the students themselves prior to the survey. Inclusion criteria required students to be in the selected classes with valid consent, while exclusion criteria included students who were unwilling to participate or unable to complete the questionnaire independently due to severe cognitive impairments.

### 2.2 Health literacy questionnaire–Chinese rapid health literacy questionnaire for elementary school students

To account for variations in cognitive development and comprehension abilities across different age groups, we developed the Chiese Rapid Health Literacy Questionnaire (CRHLQ) comprising three sub-questionnaires: CRHLQ-12 for grades 1–2, CRHLQ-34 for grades 3–4, and CRHLQ-56 for grades 5–6. Although specific items varied to ensure developmental appropriateness and avoid floor/ceiling effects, all sub-questionnaires were strictly designed to measure the same four dimensions within HL framework: Health Knowledge, Health Motivation, Health Skills, and Health Participation. The sub-questionnaires share a uniform structure comprising two sections: (1) a general information section, which collects basic characteristics on height, weight, self-assessments of health status, academic performance, and character, and (2) a measurement section assessing four HL dimensions. Specifically, Health Knowledge and Health Skills measurements consist of objective judgment or choice questions scored based on correctness (correct = 1, incorrect = 0). Health Motivation and Health Participation measurements utilize Likert-type scales—a 3-point scale for Grades 1–4 and a 5-point scale for Grades 5–6—to capture attitudes and behavioral frequencies. Final scores were calculated by summing item responses, with negative items reverse-coded prior to aggregation. Missing data were handled using listwise deletion to ensure data quality. Detailed item wording, scoring protocols, and dimensional mapping for the CRHLQ are provided in Supplementary Tables 1–3.

The total score for each sub-questionnaires is 100. Dimension weights were assigned based on importance ratings provided by a Delphi expert panel, with scores allocated accordingly. The validity and reliability of the CRHLQ was evaluated using the Rasch model of Item Response Theory (IRT). As shown in Appendix Table 6, the item reliability coefficients for all sub-questionnaires were exceptional (ranging from 0.98 to 1.00), and the item separation indices ranged from 7.72 to 15.43, far exceeding the recommended threshold of 3.0. This indicates a highly stable item hierarchy. In terms of construct validity, the Rasch dimension explained 30.2% to 59.7% of the variance, supporting the assumption of unidimensionality (see Appendix Table 6). Furthermore, the model fit was well; the mean Infit and Outfit Mean Square (MNSQ) values for items were highly consistent with the expected value of 1.0 (ranging from 0.87 to 1.00), with ZSTD scores generally falling within the ± 2.0 acceptable range (see Appendix Table 5). Although the person separation indices (1.08–1.20) and reliability coefficients (0.54–0.59) were relatively modest, the exceptional item fit statistics and high item reliability provide robust evidence of the instrument’s construct validity and measurement stability.

### 2.3 Data analysis

Descriptive statistics and Pearson correlation analyses were conducted using IBM SPSS V.29 to explore the relationships among Health Knowledge, Health Motivation, Health Skills, and Health Participation. To rigorously address the potential clustering effects arising from the multistage sampling design (students nested within regions), we first calculated the Intraclass Correlation Coefficients (ICCs) for the outcome variable (Health Participation) using linear mixed models. The results indicated that the clustering effects were minimal to negligible across all grade levels (ICC = 0.003 for Grades 1–2, 0.029 for Grades 3–4, and < 0.001 for Grades 5–6). Although the design effect was small, to ensure the most robust statistical inference, we adopted a fixed-effects approach by including regional dummy variables as covariates in all regression models to control for unobserved regional heterogeneity. Furthermore, we utilized HC3 heteroscedasticity-consistent standard errors to strictly adjust for any potential non-independence of error terms. The chain-mediated effects were tested using the PROCESS macro for SPSS (Version 4.1) developed by Andrew F. Hayes. Specifically, Model 6 was selected because it is explicitly designed to test serial (chain) mediation models. This allowed us to examine the sequential theoretical pathways and determine whether the influence of the independent variable is transmitted through a specific sequence of mediators. The significance of indirect effects was assessed using a bias-corrected non-parametric percentile bootstrap method with 5,000 replications. Effects were considered statistically significant if the 95% confidence intervals (CI) did not include zero. To account for developmental differences, all analyses were conducted separately for three grade levels (Grades 1–2, 3–4, and 5–6). Gender, academic performance, and personality traits were included as covariates in all models alongside the regional fixed effects to control for potential confounding factors. Finally, a sensitivity analysis was performed by re-estimating the models without regional fixed effects to confirm that the directions and significance of the core mediation findings remained consistent regardless of model specification.

**Figure 1:**
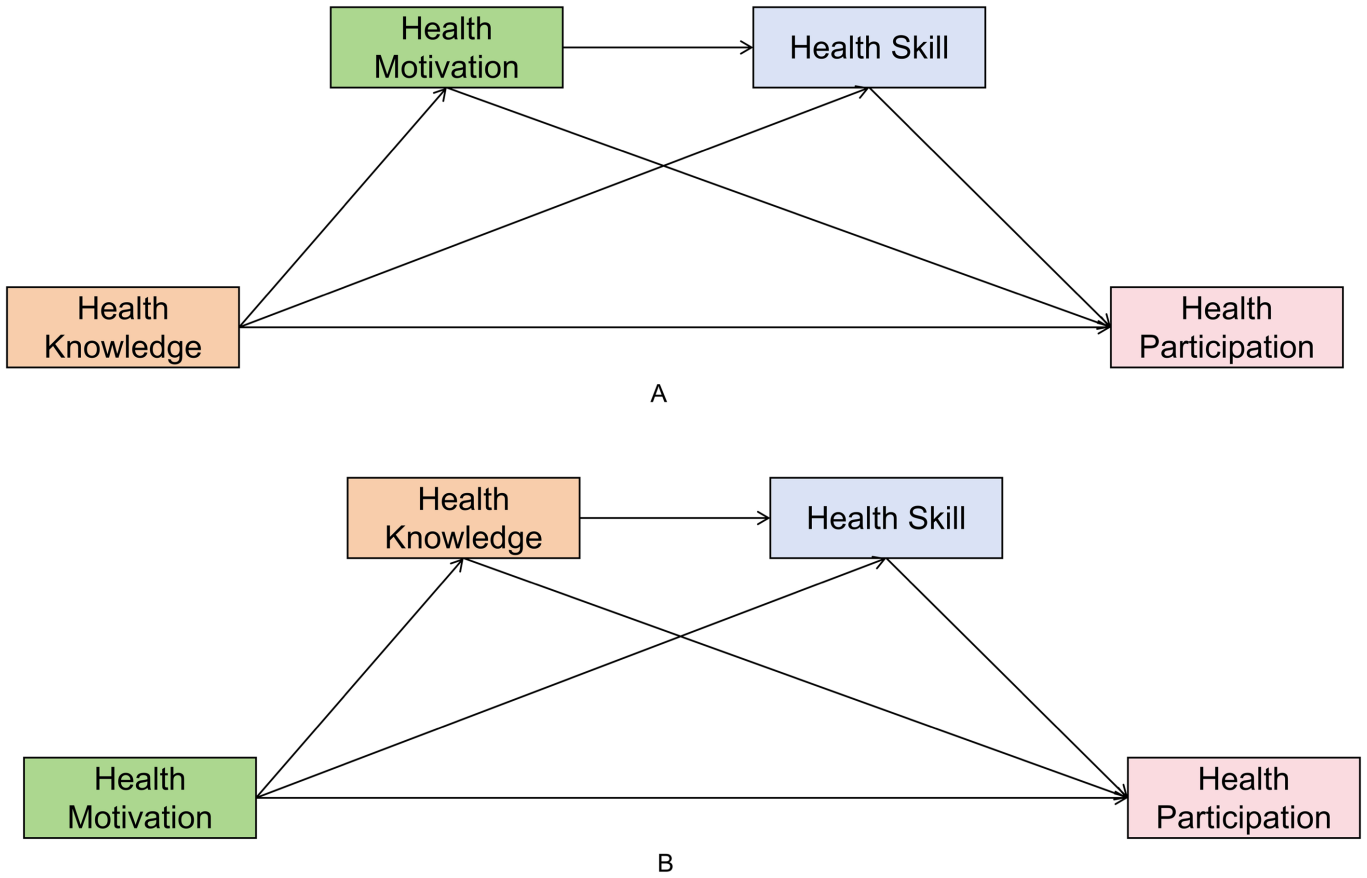
The conceptual frameworks of the two competing pathways. **(A)** Pathway 1: Knowledge-driven model; **(B)** Pathway 2: Motivation-driven model.

Two competing pathway models were constructed to examine the mechanisms driving Health Participation (Figure 1):

Pathway 1 (Knowledge-Driven): Health Knowledge → Health Motivation → Health Skill → Health Participation.

Model I tested the effect of HK on HM.

Model II tested the effects of HK and HM on HS.

Model III tested the combined effects of HK, HM, and HS on HP.

Pathway 2 (Motivation-Driven): Health motivation → Health knowledge → Health skill → Health Participation.

Model I tested the effect of HM on HK.

Model II tested the effects of HM and HK on HS.

Model III tested the combined effects of HM, HK, and HS on HP.

## 3 Result

### 3.1 Characteristics of participation

In this survey, 1,070 students were in grades 1–2, 1,190 in grades 3–4, and 1,065 in grades 5–6. The gender ratio of boys to girls was nearly equal: 47.38% girls and 52.62% boys in grades 1–2; 46.05% girls and 53.95% boys in grades 3–4; and 48.26% girls and 51.74% boys in grades 5–6. For grades 1–2 and grades 3–4, there were significant differences in HL across academic performance and character (*p* < 0.05). For grades 5--6, there were significant differences in HL by region, gender, academic performance, and character (*p* < 0.05). Details showed in Table 1.

**Table 1:** Characteristics and univariate analysis of health literacy composite scores.

Characteristic	Grades 1–2		Grades 3–4		Grades 5–6	
	N (%)	Mean±SD	N (%)	Mean±SD	N (%)	Mean±SD
Gender						
Female	507 (47.38)	86.85 ± 6.66	548 (46.05)	77.84 ± 7.20	514 (48.26)	80.75 ± 8.16
Male	563 (52.62)	86.42 ± 6.92	642 (53.95)	77.03 ± 7.70	551 (51.74)	79.16 ± 10.00
*t*	1.032		1.879		2.826	
*p*	0.302		0.060		0.005	
Nation						
Han	1,047 (97.85)	86.85 ± 6.77	1,170 (98.32)	77.40 ± 7.40	1,034 (97.46)	79.99 ± 9.16
Minority	23 (2.15)	85.39 ± 8.09	20 (1.68)	77.78 ± 11.62	28 (2.64)	77.71 ± 10.09
*t*	0.876		−0.225		1.292	
*p*	0.381		0.822		0.197	
Academic performance						
Poor	31 (2.90)	83.53 ± 5.59	60 (5.04)	72.75 ± 8.20	70 (6.60)	73.84 ± 14.28
General	785 (73.36)	86.06 ± 6.83	886 (74.45)	77.05 ± 7.45	770 (72.57)	79.51 ± 8.65
Perfect	254 (23.74)	88.71 ± 6.36	244 (20.50)	79.81 ± 6.61	222 (20.92)	83.28 ± 7.62
*F*	18.448		26.267		32.776	
*p*	<0.001		<0.001		<0.001	
Character						
Introvert	469 (43.83)	85.92 ± 6.64	566 (47.56)	76.90 ± 8.00	450 (42.41)	79.11 ± 9.97
Extrovert	601 (56.17)	87.16 ± 6.88	624 (52.44)	77.86 ± 6.95	612 (57.68)	80.53 ± 8.53
*t*	−2.976		−2.230		−2.484	
*p*	0.003		0.026		0.013	

### 3.2 Regression analysis and chain mediation test of health knowledge on health participation

**Figure 2:**
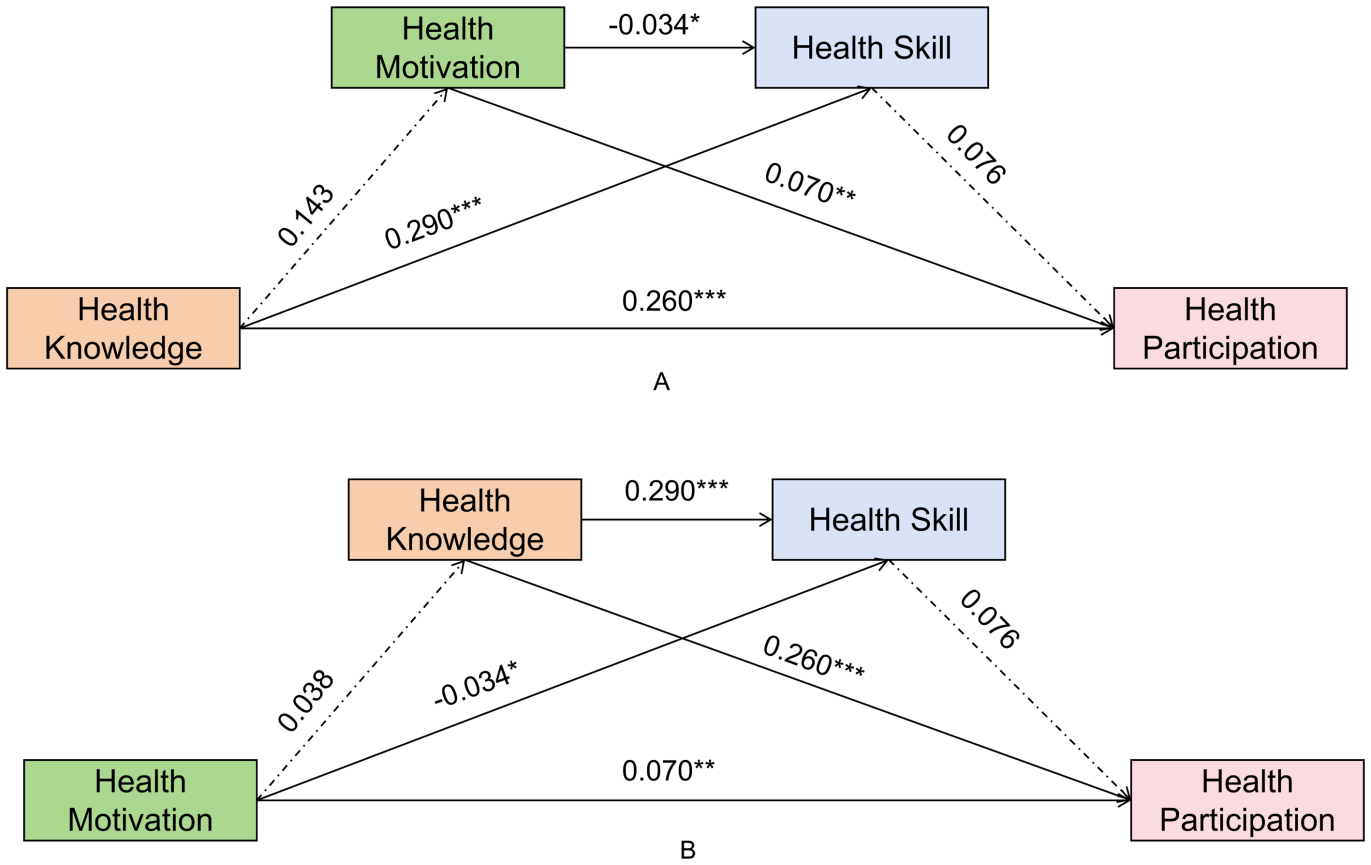
Standardized path coefficients for students in Grades 1–2. **(A)** Pathway 1: Knowledge-driven model; **(B)** Pathway 2: Motivation-driven model.

For students in Grades 1–2, the regression analysis indicated that Health Knowledge significantly predicted Health Motivation (*β* = 0.289, *p* < 0.001). Both Health Knowledge (*β* = 0.440, *p* < 0.001) and Health Motivation (*β* = 0.311, *p* < 0.001) significantly predicted Health Skill. In Model III, Health Motivation (*β* = 0.180, *p* = 0.002) and Health Skill (*β* = 0.104, *p* = 0.006) significantly predicted Health Participation, whereas the direct effect of Health Knowledge was not significant (*β* = −0.039, *p* = 0.644). Bootstrap analysis confirmed a significant total indirect effect (Effect = 0.107, 95% CI [0.053, 0.172]), and all specific indirect paths were significant (Figure 2A).

**Figure 3:**
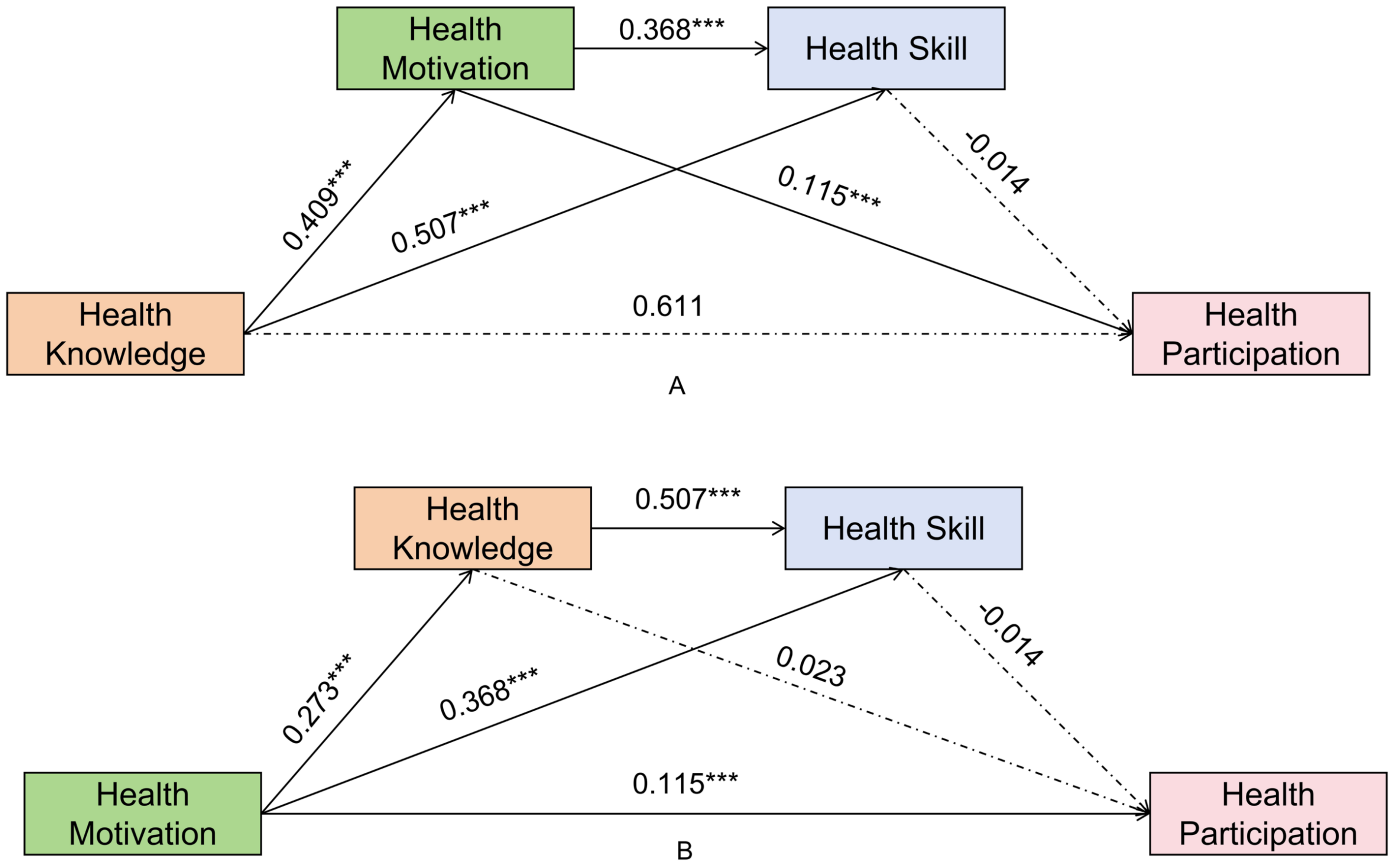
Standardized path coefficients for students in Grades 3–4. **(A)** Pathway 1: Knowledge-driven model; **(B)** Pathway 2: Motivation-driven model.

For students in Grades 3–4, the regression analysis showed that Health Knowledge did not significantly predicted Health Motivation (*β* = 0.143, *p* = 0.077). While Health Knowledge strongly predicted Health Skill (*β* = 0.290, *p* < 0.001), Health Skills did not significantly predict Health Participation (*β* = 0.076, *p* = 0.122). Instead, Health Knowledge exerted a strong and significant direct effect on Health Participation (*β* = 0.260, *p* < 0.001). Consequently, the chain mediation path was not significant [Effect = 0.000, 95% CI (−0.001, 0.000)] (Figure 3A).

**Figure 4:**
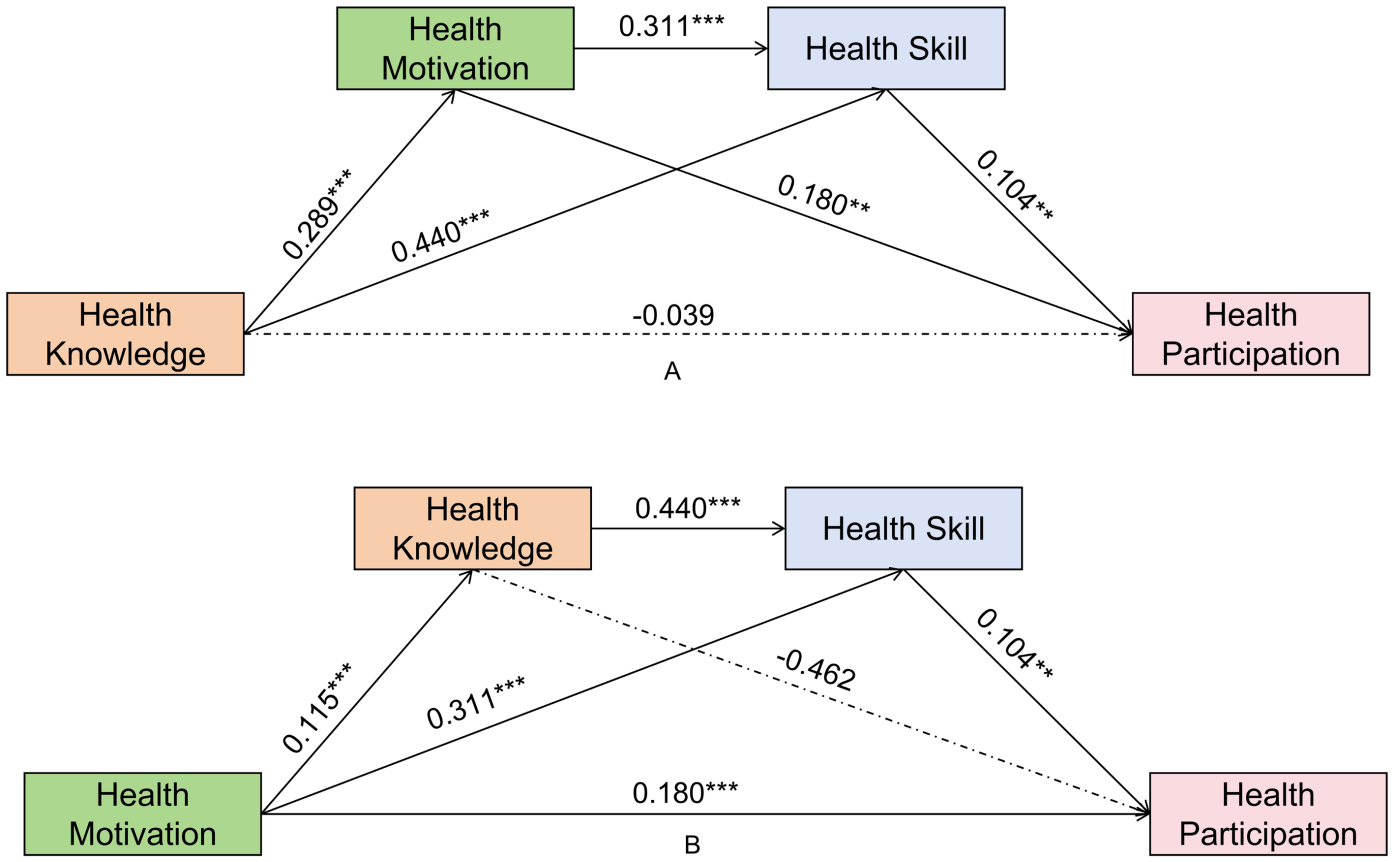
Standardized path coefficients for students in Grades 5–6. **(A)** Pathway 1: Knowledge-driven model; **(B)** Pathway 2: Motivation-driven model.

For students in Grades 5–6, the regression analysis showed Health Knowledge strongly predicted both Health Motivation (*β* = 0.409, *p* < 0.001) and Health Skill (*β* = 0.507, *p* < 0.001). Health Motivation significantly predicted Health Participation (*β* = 0.115, *p* < 0.001). The direct effect of Health Knowledge on Health Participation was not significant, but the indirect path through Motivation alone (Ind1) was significant [Effect = 0.047, 95% CI (0.028, 0.070)] (Figure 4A; Details see Tables 2, 3).

**Table 2:** Regression analysis of chain mediation effects of health knowledge, health motivation, and health skills across different grades.

Category	Dimension	Model I			Model II			Model III		
		*β*	*t*	*p*	*β*	*t*	*p*	*β*	*t*	*p*
Grade 1–2	Health Knowledge	0.289	5.332	<0.001	0.440	5.768	<0.001	−0.039	−0.462	0.644
	Health Motivation				0.311	5.909	<0.001	0.180	3.045	0.002
	Health Skill							0.104	2.776	0.006
	R	0.199			0.387			0.272		
	R^2^	0.040			0.150			0.074		
	*F* (HC3)	4.795		<0.001	18.458		<0.001	6.823		<0.001
Grade 3–4	Health Knowledge	0.143	1.771	0.077	0.290	8.491	<0.001	0.260	4.374	<0.001
	Health Motivation				−0.034	−2.113	0.035	0.070	2.616	0.009
	Health Skill							0.076	1.548	0.122
	*R*	0.140			0.374			0.315		
	*R^2^*	0.020			0.140			0.099		
	*F* (HC3)	2.904		0.003	20.542		<0.001	12.443		<0.001
Grade 5–6	Health Knowledge	0.409	9.766	<0.001	0.507	10.345	<0.001	0.023	0.611	0.541
	Health Motivation				0.368	7.566	<0.001	0.115	4.688	<0.001
	Health Skill							−0.014	−0.785	0.433
	*R*	0.355			0.526			0.244		
	*R^2^*	0.126			0.277			0.060		
	*F* (HC3)	14.009		<0.001	33.708		<0.001	5.015		<0.001

Models were adjusted for gender, academic performance, character, and regional fixed effects (provinces); HC3 robust standard errors were used to account for heteroscedasticity and clustering.

**Table 3:** Decomposition of total, direct, and indirect effects of health knowledge on health participation across different grades.

Categories	Model	Effect	Boot SE	95% Boot CI
Grades 1–2	Total effect of HK on HP	0.068		
	Total direct effect of HK on HP	−0.039	0.085	(−0.206,0.128)
	Total indirect effect of HK on HP	0.107	0.030	(0.053,0.172)
	HK → HM → HP (Ind1)	0.052	0.020	(0.018,0.096)
	HK → HS → HP (Ind2)	0.046	0.019	(0.012,0.086)
	HK → HM → HS → HP (Ind3)	0.009	0.005	(0.002,0.020)
Grades 3–4	Total effect of HK on HP	0.292		
	Total direct effect of HK on HP	0.260	0.060	(0.144,0.377)
	Total indirect effect of HK on HP	0.032	0.017	(0.001,0.067)
	HK → HM → HP (Ind1)	0.010	0.008	(0.000,0.0329)
	HK → HS → HP (Ind2)	0.022	0.015	(−0.005,0.052)
	HK → HM → HS → HP (Ind3)	0.000	0.000	(−0.001,0.000)
Grades 5–6	Total effect of X on HP	0.061		
	Total direct effect of HK on HP	0.023	0.038	(−0.051,0.098)
	Total indirect effect of HK on HP	0.038	0.014	(0.012,0.065)
	HK → HM → HP (Ind1)	0.047	0.011	(0.028,0.070)
	HK → HS → HP (Ind2)	−0.007	0.009	(−0.026,0.010)
	HK → HM → HS → HP (Ind3)	−0.002	0.003	(−0.008,0.003)

Gender, grade, academic performance, and Character are covariates. CI, Confidence Interval (Bias-corrected percentile method based on 5,000 bootstrap samples).

### 3.3 Regression analysis and chain mediation test of health motivation on health participation

For students in Grades 1–2, the regression analysis showed that Health Motivation significantly positively predicted both Health Knowledge (*β* = 0.115, *p* < 0.001) and Health Skill (*β* = 0.311, *p* < 0.001). Health Knowledge also significantly predicted Health Skill (*β* = 0.440, *p* < 0.001). Both Health Motivation (*β* = 0.180, *p =* 0.002) and Health Skill (*β* = 0.104, *p* = 0.006) significant predicted Health Participation. The bootstrap analysis confirmed the chain mediation path Health Motivation- Health Knowledge- Health Skill-Health Participation was significant [Effect = 0.005, 95% CI (0.001, 0.011)] (Figure 2B).

For students in Grades 3–4, Health Motivation did not significantly predict Health Knowledge (*β* = 0.038, *p* = 0.073). Although Health Motivation had a significant direct effect on Health Participation (*β* = 0.070, *p* = 0.009). The path Health Motivation- Health Knowledge- Health Participation was not significant [Effect = 0.001, 95% CI (0.000, 0.0003)] (Figure 3B).

For students in Grades 5–6, the regression analysis showed that Health Motivation strongly predicted Health Knowledge (*β* = 0.273, *p* < 0.001) and Health Knowledge strongly predicted Health Skill (*β* = 0.507, *p* < 0.001). Neither Health Knowledge (*β* = 0.023, *p* = 0.541) nor Health Skill (*β* = −0.014, *p* = 0.433) significantly predicted Health Participation in Model III. The impact of Health Motivation on Health Participation was primarily driven by a significant direct effect (*β* = 0.115, *p* < 0.001). All indirect paths, including the chain mediation, were not significant (Figure 4B; Details see Tables 4, 5).

**Table 4:** Regression analysis of chain mediation effects of health motivation, health knowledge and health skills across different grades.

Category	Dimension	Model I			Model II			Model III		
		*β*	*t*	*p*	*β*	*t*	*p*	*β*	*t*	*p*
Grade 1–2	Health Motivation	0.115	4.476	<0.001	0.311	5.909	<0.001	0.180	3.045	0.002
	Health Knowledge				0.440	5.768	<0.001	−0.039	−0.462	0.644
	Health Skill							0.104	2.776	0.006
	R	0.208			0.387			0.272		
	R^2^	0.043			0.150			0.074		
	*F* (HC3)	4.113		<0.001	18.458		<0.001	6.823		<0.001
Grade 3–4	Health Motivation	0.038	1.795	0.073	−0.034	−2.113	0.035	0.070	2.616	0.009
	Health Knowledge				0.290	8.491	<0.001	0.260	4.374	<0.001
	Health Skill							0.076	1.548	0.122
	R	0.215			0.374			0.315		
	R^2^	0.046			0.140			0.099		
	*F* (HC3)	9.618		<0.001	20.542		<0.001	12.443		<0.001
Grade 5–6	Health Motivation	0.273	7.704	<0.001	0.368	7.566	<0.001	0.115	4.688	<0.001
	Health Knowledge				0.507	10.345	<0.001	0.023	0.611	0.541
	Health Skill							−0.014	−0.785	0.433
	R	0.389			0.526			0.244		
	R^2^	0.152			0.277			0.060		
	*F* (HC3)	12.054		<0.001	33.708		<0.001	5.015		<0.001

Models were adjusted for gender, academic performance, character, and regional fixed effects (provinces); HC3 robust standard errors were used to account for heteroscedasticity and clustering.

**Table 5:** Decomposition of total, direct, and indirect effects of health motivation on health participation across different grades.

Categories	Model	Effect	Boot SE	95% Boot CI
Grade 1–2	Total effect of HM on HP	0.213		
	Total direct effect of HM on HP	0.180	0.059	(0.064,0.296)
	Total indirect effect of HM on HP	0.033	0.016	(0.002,0.066)
	HM → HK → HP (Ind1)	−0.005	0.010	(−0.026,0.015)
	HM → HS → HP (Ind2)	0.032	0.013	(0.009,0.060)
	HM → HK → HS → HP (Ind3)	0.005	0.003	(0.001,0.011)
Grade 3–4	Total effect of HM on HP	0.078		
	Total direct effect of HM on HP	0.070	0.027	(0.017,0.122)
	Total indirect effect of HM on HP	0.008	0.007	(−0.004,0.024)
	HM → HK → HP (Ind1)	0.010	0.006	(0.000,0.024)
	HM → HS → HP (Ind2)	−0.003	0.002	(−0.008,0.001)
	HM → HK → HS → HP (Ind3)	0.001	0.001	(0.000,0.003)
Grade 5–6	Total effect of HM on HP	0.114		
	Total direct effect of HM on HP	0.115	0.025	(0.067,0.164)
	Total indirect effect of HM on HP	−0.001	0.012	(−0.024,0.025)
	HM → HK → HP (Ind1)	0.006	0.010	(−0.012,0.030)
	HM → HS → HP (Ind2)	−0.005	0.007	(−0.019,0.008)
	HM → HK → HS → HP (Ind3)	−0.002	0.003	(−0.007,0.003)

Gender, grade, academic performance, and Character are covariates. CI, Confidence Interval (Bias-corrected percentile method based on 5,000 bootstrap samples).

## 4 Discussion

This study examines two pathways of health participation improvement across different school stages. Results showed that pathways exhibited a motivation-driven pattern in lower grades, a knowledge-driven pattern in middle grades, and a combined knowledge- and motivation-driven pattern in upper grades. We also assessed HL levels and examined associated factors, supporting prior studies that academic performance, gender, and personality traits are associated with HL levels (24–26).

Young children depend more on willingness to participate in health behaviors because their cognitive skills are still developing, making knowledge alone insufficient to encourage action. Middle grades likely represent a knowledge-sensitive window, where understanding significantly enhances the explanatory power of health knowledge. In upper grades, students may integrate both knowledge and motivation into self-regulated decision-making, enabling them to sustain health behaviors more independently. These align with the previous evidence that autonomy, perceived competence, and intrinsic motivation become more influential in preadolescence (27, 28).

Our findings underscore the critical, yet often overlooked, role of motivation in health promotion for children. While the literature on HL and school-based interventions is extensive, examining diverse aspects such as knowledge acquisition (29), recognition and willingness (30), motivation (31), and skill-building (32), the dominant paradigm for elementary students remains distinctly knowledge-driven. This is evidenced by many RCTs focusing primarily on imparting knowledge in areas such as food hygiene (33), nutrition (34), oral health (35), mental health (36), disease prevention (37), and healthy decision-making (38). One meta-analysis synthesized evidence of high-quality trials examining training workshops and digital resources on health choices-related critical thinking for lower secondary school students (39). In contrast, the potential of a systematic motivation-driven approach has been overwhelmingly under-investigated. Although self-efficacy interventions show promise (40–42).

Framework-based interventions are essential for effective health education. The field employs diverse guiding frameworks, ranging from specific theories of health behavior change, such as the Information-Motivation-Behavioral Skills model (43), and Social Cognitive Theory (31), to broader frameworks for intervention development, such as the Medical Research Council framework (44). However, few of these theory-based interventions have been developed and evaluated for elementary school students. Given the rapid cognitive and developmental evolution during these years, theory-based and approach-oriented interventions are crucial for effective implementation. Furthermore, the rigorous evaluation of such tailored interventions depends on robust and comprehensive HL assessment tools to accurately track outcomes and measure impact.

Crucially, research on framework-based HL education approaches remains scarce, particularly for elementary students. This study addresses this gap by providing a direct comparative analysis of two key pathways, highlighting the need for a motivation-driven approach in the lower grades. The findings offer an evidence-based reference for tailoring school-based health education. For effective implementation, teacher training should be aligned with these developmental stages. For Grades 1–2, training could equip teachers to prioritize motivation and procedural skills through play-based activities, modeling, and guided practice. For Grades 3–4, the focus could shift to developing teachers’ ability to deliver structured instruction that fosters conceptual understanding and rule-based reasoning. For Grades 5–6, training should emphasize autonomy-supportive methods, such as facilitating goal setting, reflective learning, and self-management strategies, to help students internalize motivation and build self-regulation.

## 5 Limitations

First, the cross-sectional design precludes definitive causal inference; thus, the identified pathways represent does not support confirmed causality. Second, due to data constraints and the sampling structure, specific comparative analyses between urban and rural areas could not be conducted in this study. Finally, the modest Rasch person reliability coefficients indicate limited sensitivity for individual-level discrimination, though the instrument remains valid for the group-level structural analyses conducted here.

## 6 Conclusion

Guided by the HL framework, this study systematically examined two key improvement pathways: knowledge-driven and motivation-driven. Our findings reveal distinct, grade-specific patterns in health participation improvement, indicating that a motivation-driven approach is critical in the lower grades. It is essential to implement framework-based and approach-appropriate HL interventions and educational strategies for elementary school students.

## Data Availability

The raw data supporting the conclusions of this article will be made available by the authors, without undue reservation.
