# Supplementary material for: Knowledge-driven or motivation-driven? Validation and comparison of health participation pathways across different elementary grade levels
Source: Front Public Health. 2026 Feb 18;14:1772064. doi: 10.3389/fpubh.2026.1772064 (PMC12956628; doi:10.3389/fpubh.2026.1772064)
Supplement: Supplementary file 1 [file Data_Sheet_1.docx]

Supplementary Material

**Supplementary Figure 1.** Items of CRHLQ-12

| Item | Score | Question Type | Dimension |
| --- | --- | --- | --- |
| B1 Adequate sleep can eliminate fatigue and improve learning efficiency. | 2 | Judgement Question  Judgement Question | Health Knowledge |
| B2 Being able to get along friendly with peers is a sign of mental health. | 2 |  |  |
| B3 When walking, one should be cautious of vehicles parked by the roadside suddenly starting. | 2 |  |  |
| B4 Frequently eating sweet foods and drinking sugary beverages can easily cause dental caries, which is also known as tooth decay. | 2 |  |  |
| B5 One should wash hands before each meal and after using the restroom. | 2 |  |  |
| B6 A reasonable diet means eating more meat and less vegetables. | 2 |  |  |
| B7 Practicing good hygiene, washing hands frequently, ventilating rooms, and eating clean food can reduce the risk of infectious diseases. | 2 |  |  |
| B8 During an earthquake, one can take the elevator to escape. | 2 |  |  |
| B9 Children should exercise for at least one hour every day. | 2 |  |  |
| B10 It doesn’t matter if the eyes are nearsighted, as it can be cured. | 2 |  |  |
| B11 If food has just expired recently, it can continue to be eaten. | 2 |  |  |
| B12 Among the three sports of football, badminton, and table tennis, I can play at least one. | 2 |  |  |
| C1 Is Xiaoming’s statement correct? | 2 |  | Health Skill |
| C2 A few days later, Xiaohua returned to school and wore a mask. Was his way of wearing it correct? | 2 |  |  |
| C3 Whose approach was correct, Ningning’s mother or the doctor on the show? | 2 |  |  |
| C4 The doctor on the show said that using less oil is beneficial for health. What are the benefits of eating less oil? | 2 |  |  |
| C5 If you suddenly feel nauseous and have a stomachache after eating cold tremella salad at noon, what do you think might be the reason? | 2 |  |  |
| C6 When you feel in a bad mood, which of the following practices is incorrect? | 3 |  |  |
| C7 If you encounter a bad person, which of the following phone numbers should you call for help? | 3 |  |  |
| C8 What do you think is the correct way to wash hands? | 3 |  |  |
| C9 When you are thirsty, which drink is healthier for quenching thirst? | 3 |  |  |
| C10 Xiaoming had milk, steamed buns, and an apple for breakfast but still felt hungry. What else should he eat for a more reasonable and healthy dietary nutrition balance? | 3 |  |  |
| D1 I like to do things that are beneficial for health. | 3 | 3-point Likert-type | Health Motivation |
| D2 Good health improves my disease resistance. | 3 |  |  |
| D3 My body is very healthy, so I don’t need to consider health-related matters. | 3 |  |  |
| D4 I want to take responsibility for my own life. | 3 |  |  |
| D5 I want to get sick less often. | 3 |  |  |
| D6 Academic performance is more important than health; being unhealthy is okay. | 3 |  |  |
| E1 Lack confidence in oneself, believing to be inferior in all aspects. | 3 |  | Health Participant |
| E2 Face setbacks and failures bravely without discouragement. | 3 |  |  |
| E3 Play alone at heights (windowsills, balconies, roofs, or rockeries). | 3 |  |  |
| E4 Pet, kiss, or hug stray cats, dogs, and other unfamiliar animals. | 3 |  |  |
| E5 Sleep 10 hours every day. | 3 |  |  |
| E6 Brush teeth in the morning and evening. | 3 |  |  |
| E7 Eat directly without washing hands even when hands look clean. | 3 |  |  |
| E8 Immediately tell teachers or parents when feeling unwell. | 3 |  |  |
| E9 Take the initiative to wear a mask in the classroom during the high-incidence season of infectious diseases. | 3 |  |  |
| E10 Read books while lying in bed. | 3 |  |  |
| E11 Eat food sold at roadside stalls. | 3 |  |  |

**Supplementary Table 2。** Items of CRHLQ-34

| Item | Score | Question Type | Dimension |
| --- | --- | --- | --- |
| B1 Eye fatigue and involuntary eye-rubbing are normal; just pay attention to resting. | 2 | Judgement Question | Health Knowledge |
| B2 Partiality for certain foods can cause nutritional imbalance. | 2 |  |  |
| B3 Red date-flavored yogurt may contain "added sugar." | 2 |  |  |
| B4 Housework and sports both count as physical activities. | 2 |  |  |
| B5 It’s okay to sit and study without moving for a whole morning. | 2 |  |  |
| B6 Adequate sleep can eliminate fatigue and improve learning efficiency. | 2 |  |  |
| B7 Expired food can still be eaten if it doesn’t taste different. | 2 |  |  |
| B8 Being able to get along friendly with peers is one sign of mental health. | 2 |  |  |
| B9 Frequently eating sweets like chocolate, cakes, and candies can easily cause dental caries. | 2 |  |  |
| B10 The transmission routes of influenza include airborne droplets. | 2 |  |  |
| B11 Among football, badminton, and table tennis, I can play at least one. | 2 |  |  |
| C1 Which of the following behaviors is incorrect for earthquake self-rescue? | 2 | Choice Question | Health Skill |
| C2 What do you think is the correct way to wash hands? | 2 |  |  |
| C3 What is the best thing to do when you feel upset or encounter problems you can’t solve? | 2 |  |  |
| C4 Which of the following is a wrong way to brush teeth? | 2 |  |  |
| C5 The thermometer shows ___, and this temperature is ___. | 2 |  |  |
| C6 If you have a stomachache after eating, which part of the body (in the picture) is hurting? | 2 |  |  |
| C7 Which food set is healthier when you eat at a restaurant? | 2 |  |  |
| D1 I like to do things beneficial for health. | 1 | 3-point Likert-type | Health Motivation |
| D2 Health aligns with my life goals. | 3 |  |  |
| D3 Good health enhances my disease resistance. | 3 |  |  |
| D4 I maintain health because parents and teachers require it. | 3 |  |  |
| D5 My body is in good condition, so I don’t need to think about health-related matters. | 3 |  |  |
| D6 I feel a sense of satisfaction from maintaining health through my own efforts. | 3 |  |  |
| D7 I want to take responsibility for my life. | 3 |  |  |
| D8 Health keeps me in a better state. | 3 |  |  |
| D9 I want to be more energetic. | 3 |  |  |
| D10 I want to get sick less often. | 3 |  |  |
| D11 I maintain health because everyone says it’s important. | 3 |  |  |
| D12 Academic performance is more important than health; being unhealthy is acceptable. | 3 |  |  |
| E1 Play alone at high places like platforms, terraces, or roofs. | 3 |  | Health Participant |
| E2 Pet, kiss, or hug stray cats, dogs, and other unfamiliar animals. | 3 |  |  |
| E3 Sleep for 10 hours. | 3 |  |  |
| E4 Wash hands 自觉 (voluntarily) before meals and after using the restroom. | 3 |  |  |
| E5 Brush teeth twice a day. | 3 |  |  |
| E6 Check the production date and shelf life carefully before buying food. | 3 |  |  |
| E7 Buy street food from mobile vendors. | 3 |  |  |
| E8 Get along friendly with classmates and communicate actively. | 3 |  |  |
| E9 Face setbacks and failures without discouragement. | 3 |  |  |
| E10 Take the initiative to wear a mask in the classroom during infectious disease outbreaks. | 3 |  |  |

**Supplementary Table 3.**  Items of CRHLQ-56

| Item | Score | Question Type | Dimension |
| --- | --- | --- | --- |
| B1 Adequate sleep provides us with a good physical state and improves learning efficiency. | 1 | Judgement Question | Health Knowledge |
| B2 Mental health is an important part of overall health, and physical and mental health are closely linked and influence each other. | 1 |  |  |
| B3 Being sociable, adapting to group life, and getting along friendly with peers are among the signs of mental health for primary and secondary school students. | 2 |  |  |
| B4 Expressing emotions actively and communicating can release psychological pressure and alleviate negative feelings. | 2 |  |  |
| B5 Only older people can get chronic diseases. | 2 |  |  |
| B6 Carbonated beverages contain a large amount of sugar. Long-term excessive consumption can lead to sugar accumulation and nutrient excess, causing sugar to be converted into subcutaneous fat and leading to weight gain. | 2 |  |  |
| B7 After a joint contusion, one should rest immediately, stop exercising, and avoid moving the injured joint. | 2 |  |  |
| B8 Children’s bones are less strong than adults’, making them more prone to fractures after trauma. | 2 |  |  |
| B9 Pit and fissure sealing is a procedure where dentists fill the grooves on tooth surfaces to prevent bacteria and food debris from entering. | 2 |  |  |
| B10 Frequent handwashing and maintaining personal hygiene can prevent influenza. | 2 |  |  |
| B11 Drinking untreated water can cause intestinal infections (such as enteritis, dysentery, typhoid fever, etc.). | 2 |  |  |
| B12 Physical activity that lasts less than 60 minutes a day or is of low intensity is useless for health and might as well not be done. | 2 |  |  |
| B13 Prolonged sitting, watching TV, videos, playing computer games, reading, drawing, and doing homework are considered sedentary behaviors. | 2 |  |  |
| B14 A nutritious diet, adequate sleep, and active outdoor activities have no relation to eye protection. | 2 |  |  |
| C1 Ningning’s mother often says, “More oil makes dishes better,” and always adds a lot of oil to make food tastier. At 6 PM, a health program on TV states that using less oil in meals is beneficial for health. | 2 | Choice Question | Health Skill |
| C2 Summer and autumn are peak seasons for digestive tract infections. What are the typical symptoms of digestive tract infections? | 2 |  |  |
| C3 After school, you and your deskmate hesitate between milk and yogurt at the supermarket. Your deskmate buys yogurt and says, “Drinking yogurt is the same as drinking milk.” What would you do? | 2 |  |  |
| C4 Which of the following is an incorrect way to brush teeth? | 2 |  |  |
| C5 Is the practice of “cutting vegetables first before washing them” correct when preparing vegetables? | 2 |  |  |
| C6 Analyze the following behaviors: Which measures do you think can prevent diarrhea? | 2 |  |  |
| C7 What is an inappropriate measure to take when facing setbacks? | 2 |  |  |
| C8 When discovering your own psychological problems, which of the following actions is incorrect? | 2 |  |  |
| C9 What do you think is the correct way to wash hands? | 2 |  |  |
| C10 What should be done after a soft tissue contusion? | 2 |  |  |
| C11 Which of the following self-rescue behaviors is wrong in case of a fire? | 2 |  |  |
| C12 Which of the following self-rescue behaviors during an earthquake is incorrect? | 2 |  |  |
| C13 Which classmate’s statement below is wrong? | 2 |  |  |
| C14 What should you do if the food at home is too salty, oily, or sweet? | 2 |  |  |
| C15 What should you do when feeling unwell? | 2 |  |  |
| D1 I enjoy doing things that are beneficial to my health. | 2 | 5-point Likert-type | Health Motivation |
| D2 Health aligns with my life goals. | 2 |  |  |
| D3 Good health enhances my disease resistance. | 2 |  |  |
| D4 I maintain health because my parents and teachers require it. | 2 |  |  |
| D5 My body is in good condition, so I don’t need to think about health-related matters. | 2 |  |  |
| D6 I feel a sense of accomplishment from maintaining health through my own efforts. | 2 |  |  |
| D7 I want to take responsibility for my life. | 2 |  |  |
| D8 Health keeps me in a better physical and mental state. | 2 |  |  |
| D9 I want to be more energetic. | 2 |  |  |
| D10 I want to get sick less often. | 2 |  |  |
| D11 I maintain health because everyone says it’s important. | 2 |  |  |
| D12 Academic performance is more important than health; being unhealthy is acceptable. | 2 |  |  |
| E1 I stop browsing or playing when I see health-related information online. | 2 |  | Health Participant |
| E2 I obtain health knowledge about infectious diseases (such as COVID-19), food poisoning, and myopia prevention through TV, the internet, and other channels. | 2 |  |  |
| E3 I actively search for health-related knowledge like myopia prevention and oral hygiene online. | 2 |  |  |
| E4 I can get 10 hours of sleep. | 2 |  |  |
| E5 I cross the road without checking traffic lights and rush through quickly. | 2 |  |  |
| E6 I change and wash my underwear regularly. | 2 |  |  |
| E7 I eat while walking or playing. | 2 |  |  |
| E8 I eat food from roadside stalls. | 2 |  |  |
| E9 I drink carbonated beverages. | 2 |  |  |
| E10 I eat snacks. | 2 |  |  |

**Supplementary Table 4. S**core Allocation for Each Dimension of the Health Literacy Questionnaire

| Sub-questionnaire | Dimension | Score | Item |
| --- | --- | --- | --- |
| CRHLQ-12 | Health Knowledge | 24 | B1-B12 |
|  | Health Skill | 25 | C1-C10 |
|  | Health Motivation | 18 | D1-D6 |
|  | Health Practice | 33 | E1-E11 |
| CRHLQ-34 | Health Knowledge | 22 | B1-B11 |
|  | Health Skill | 14 | C1-C7 |
|  | Health Motivation | 34 | D1-D12 |
|  | Health Practice | 30 | E1-E10 |
| CRHLQ-56 | Health Knowledge | 26 | B1-B11 |
|  | Health Skill | 30 | C1-C15 |
|  | Health Motivation | 24 | D1-D12 |
|  | Health Practice | 20 | E1-E10 |

| Sub-Questionnaire | Number | Measure | SE | INFIT | | OUTFIT | |
| --- | --- | --- | --- | --- | --- | --- | --- |
|  |  |  |  | MNSQ | ZSTD | MNSQ | ZSTD |
| CRHLQ-12 | Person(n=1070) | 2.82 | 0.49 | 1.01 | 0.00 | 0.91 | 0.10 |
|  | Item(n=39) | 0.00 | 0.14 | 1.00 | 0.30 | 0.87 | -0.20 |
| CRHLQ-34 | Person(n=1190) | 1.81 | 0.39 | 0.99 | 0.00 | 1.03 | 0.00 |
|  | Item(n=40) | 0.00 | 0.09 | 0.99 | 0.40 | 0.97 | 0.10 |
| CRHLQ-56 | Person(n=1065) | 1.31 | 0.34 | 0.98 | -0.01 | 1.04 | -0.01 |
|  | Item(n=52) | 0.00 | 0.10 | 0.99 | 0.10 | 0.98 | 0.10 |

**Supplementary Table 5.** Person and Item fit summary statistics for the CRHLQ

**Supplementary Table 6.** Summary of changes in separation, reliability, and variance explained.

| Questionnaire | Separation | | Reliability | | Variance explained |
| --- | --- | --- | --- | --- | --- |
|  | Person | Item | Person | Item |  |
| CRHLQ-12 | 1.20 | 7.72 | 0.59 | 0.98 | 30.2% |
| CRHLQ-34 | 1.13 | 14.43 | 0.56 | 1.00 | 54.0% |
| CRHLQ-56 | 1.08 | 15.43 | 0.54 | 1.00 | 59.7% |
